# Morphological, Mechanical and Thermal Properties of Rubber Foams: A Review Based on Recent Investigations

**DOI:** 10.3390/ma16051934

**Published:** 2023-02-26

**Authors:** Ehsan Rostami-Tapeh-Esmaeil, Denis Rodrigue

**Affiliations:** Department of Chemical Engineering, Laval University, Quebec, QC G1V 0A6, Canada

**Keywords:** rubber, foam, foaming agent, morphology, mechanical properties

## Abstract

During recent decades, rubber foams have found their way into several areas of the modern world because these materials have interesting properties such as high flexibility, elasticity, deformability (especially at low temperature), resistance to abrasion and energy absorption (damping properties). Therefore, they are widely used in automobiles, aeronautics, packaging, medicine, construction, etc. In general, the mechanical, physical and thermal properties are related to the foam’s structural features, including porosity, cell size, cell shape and cell density. To control these morphological properties, several parameters related to the formulation and processing conditions are important, including foaming agents, matrix, nanofillers, temperature and pressure. In this review, the morphological, physical and mechanical properties of rubber foams are discussed and compared based on recent studies to present a basic overview of these materials depending on their final application. Openings for future developments are also presented.

## 1. Introduction

Rubber foams are composite materials composed of a dispersed gaseous phase surrounded by an elastomeric matrix [1]. Rubber foams have high resilience, flexibility, tear resistance, deformability (especially at low temperature), abrasion resistance, buoyancy and lower weight, making them important materials in our daily lives [2,3]. In addition, their high elasticity, strength-to-weight ratio, impact resistance and energy absorption provide them with a wide range of various applications, including in automobiles, transportation, aeronautics, building, construction and cushioning [4]. However, more applications will expand to a larger variety of conditions with more technological development, different temperatures, pressures, mechanical solicitation, radiation or environmental conditions [5].

The first cellular polymer to enter the commercial market was a rubber foam developed in 1914 [6]. It was prepared by combining a natural rubber (NR) latex with gas-producing compounds (foaming agents), such as sodium and ammonium carbonate or sodium polysulfide. The objective is to give a broad insight related to important parameters including formulation (foaming agent, matrix and nanofiller) and foaming (temperature, pressure and time) parameters on the morphological, mechanical, physical and thermal properties of rubber foams.

## 2. Structural Features

### 2.1. Porosity: Open or Closed Cell

The expansion of a gaseous phase dispersed throughout a rubber matrix is necessary to produce a rubber foam. According to the porosity and cell hole, cellular materials can be typically classified as open cells (direct connection between the gas cells/broken walls) or closed cells (gas cell are distinct/unbroken walls) [7]. Foams with an open cell structure are more prevalent in less rigid materials, while closed cells represent more rigid ones [8]. The final application will depend on structural parameters. The high porosity of open cell foams lets them be good candidates for fuel tank inserts, filtration media, medical devices, fluid management and loudspeakers, due to their lower weight and lower cost. Because of their interconnections, the cells can allow fluids (gas or liquid) to travel across, which is beneficial for applications such as filtration and separation operations. Closed cell foams are generally more expensive, having lower porosity leading to better resistance and reduced adsorption/absorption which are interesting for thermal, sound and electrical insulators. In both cases, a chemical (CFA) and/or physical (PFA) foaming agent can be used to create closed or open cell structures [9].Vahidifar et al. prepared a closed cell NR/nanoclay (NC) foam using azodicarbonamide (ADC, 4 phr) as a CFA [10]. They found that the foaming of NR/NC (0–10 phr) at 155 °C for 30 min led to a closed cell structure. Another work stated that the addition of carbon nanotube (CNT = 1–3 phr) and nanoclay (NC = 2–6 phr) with different geometry and content led to a different closed/open cell ratio inside EPDM foams [11]. The tubular structure of CNT generated closed cells at low content, while higher concentration led to higher open cell content. Nevertheless, open cell formation was restricted by the presence of organo-modified NC, which had a plate-like structure leading to lower open cell content with increasing NC content.

**Figure 1:**
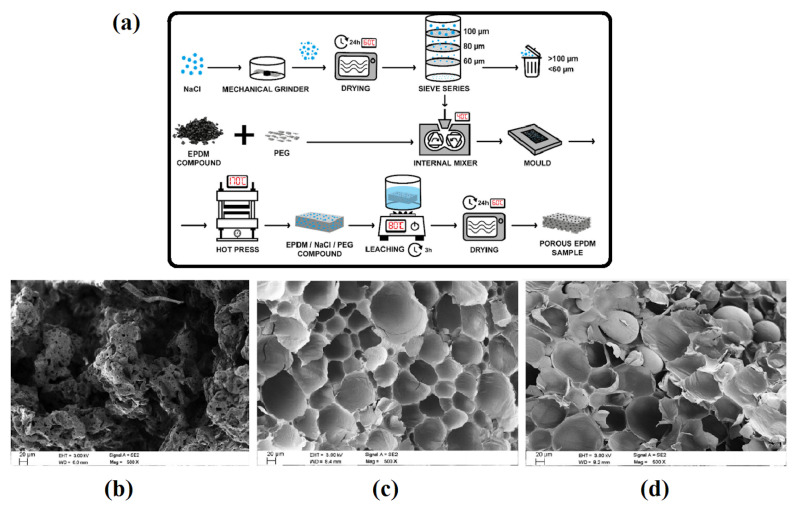
(**a**) The preparation steps for EPDM foams by the salt leaching technique and (**b**) SEM image of the resulting foam compared to foams based on (**c**) Micropearl and (**d**) Expancel [12].

Xie et al. reported on a “sea-island” structure resulting in a complex network inside NR by using expanded polymer microspheres as a foaming agent [3]. By adding 2.04 vol% CNT, they produced low density NR foams (0.50 g/cm^3^) with good thermal (0.048 W/m.K) and electrical (36.6 S/m) conductivities. They also achieved NR foams with the lowest thermal conductivity of 0.02 W/m.K. Zonta et al. compared the salt leaching technique (Figure 1a) and traditional foaming process (Micropearl and Expancel) in terms of morphology and thermal conductivity for EPDM foams [12]. The salt leaching method led to an open cell structure, while the foaming agents led to closed cells with diameters ranging between 60 and 80 μm, as reported in Figure 1b–d. They also measured the thermal conductivity of their foams and observed that the value of NaCl/EPDM foam was 36.4% and 18.2% higher than for foams using Micropearl and Expancel, respectively. Furthermore, the compression set of conventional foaming methods was 160% and 392.5% higher than for NaCl/EPDM.

### 2.2. Cell Size and Cell Density Effect

**Figure 2:**
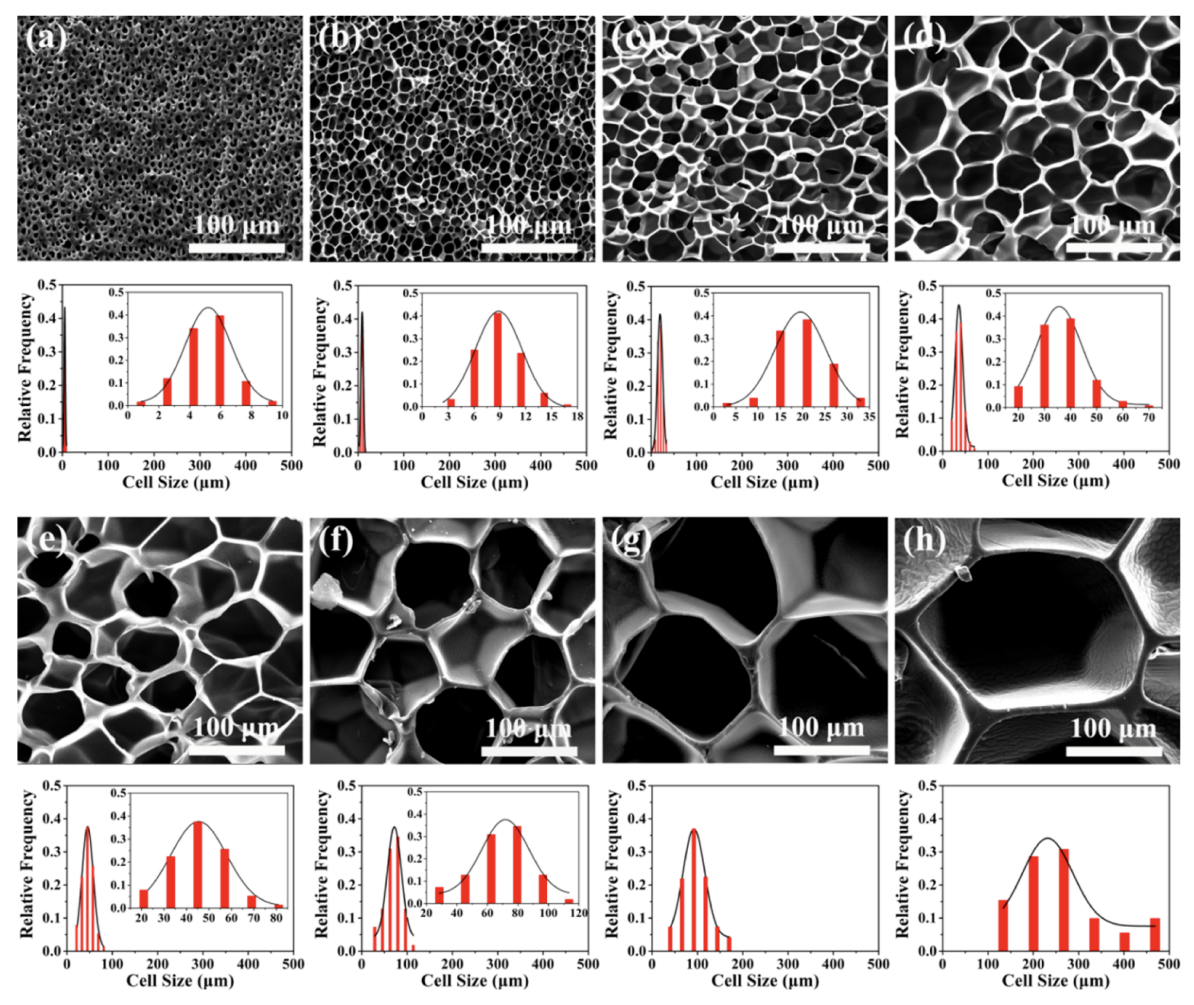
SEM images and cell morphology of SR foams designed by controlling the scCO_2_ foaming conditions with different cell sizes (µm): (**a**) 5, (**b**) 9, (**c**) 20, (**d**) 34, (**e**) 45, (**f**) 71, (**g**) 107, (**h**) 266 [16].

According to the size of the cell, rubber foams can be divided into four categories: nanocellular (0.1–100 nm), ultramicrocellular (0.1–1 µm), microcellular (1–100 μm) and macrocellular (above 100 µm) [13]. In general, nanocellular foams are described as having cells less than 100 micron with a cell density higher than 10^12^ cells/cm^3^. Nanocellular foams are expected to have several qualities superior to those of conventional foams because of their distinctive architectures (higher surface area), including a better strength-to-weight ratio, excellent filtration (separation) ability, lower dielectric constant and high thermal and sound insulation behavior [14]. Several studies have shown that the cells’ dimensions directly affect the final foam’s properties. For example, Rostami and coworkers prepared graded polyolefin elastomer (POE) foams with a range of cell size (98–337 µm) and cell density (204–820 cells/mm^3^) across the thickness [15]. Their findings confirmed that the side of the foam with lower cell sizes/higher cell density provided improved thermal resistance. Tang et al. used supercritical carbon dioxide (scCO_2_) foaming to create silicon rubber (SR) foams with various cell sizes ranging from 5 to 266 µm, leading to different cell shapes from spherical to polygonal, as presented in Figure 2 [16]. It was concluded that SR foams may be used in various applications depending on their cellular microstructures: foams with small cells (approximately 5 µm) had high compression strength, while large cells (above 50 µm) had good recovery performance and medium cell size (5–50 µm) showed good tensile properties. Syahrin et al. studied the effect of sodium bicarbonate (NaHCO_3_) concentration (3 to 12 phr) on the cell size and compression properties of NR latex foam (NRLF) [17]. They reported that the average cell diameter of NRLF decreased by 15% as the CFA content increased from 3 to 12 phr, while the compression strength decreased from 0.54 to 0.08 MPa at 3 phr compared to an increase up to 0.19 MPa at 13 phr.

Jia et al. studied the effect of expansion ratios on SR/nanographene (NG) composite foams’ morphology [18]. It was found that these foams had both open and closed cells morphologies. Moreover, the cell density was increased (2.13–7.15 × 10^5^/cm^3^), while the cell diameter decreased (191–79.4 µm) as the foaming expansion ratio increased from 2 to 5. For composites at low ER, the large cells (>150 µm) dominated (73%), while smaller cells (<150 µm) dominated (93%) at higher ER. They also observed that a higher number of smaller cells enhanced the tunneling effects, leading to lower dielectric constants. Finally, higher cell density improved the NG distribution (more uniform), which also improved the dielectric constant.

## 3. Foaming Process

The foaming and crosslinking reactions are two opposing reactions that play a significant role in controlling the final foam properties by influencing the cell morphology during the rubber foam production. While the curing reaction between rubber molecules stabilizes the morphology as well as increases the viscosity, the gas generated via CFA decomposition leads to foaming. Gas molecules inside the rubber matrix decrease the curing characteristics (e.g., curing rate, torque, scorch time, etc.). They act as a solvent and aggregate between the macromolecule chains of the matrix, leading to higher free volume and chain mobility. On the other hand, the presence of foaming agents causes the system to absorb more heat, which inhibits the crosslinking process. During the decomposition of the blowing agent, the gas expands and decreases the crosslink density.

The foaming and curing processes can be effectively managed by selecting appropriate processing parameters, including temperature and formulation (components and concentration). To manage the final foam cellular structure (cell size and cell density), the kinetics of these two reactions must be balanced. When the pressure is released or the foaming agent begins to decompose and releases its gas, if the rubber has slow curing and is not adequately pre-cured, the gas will either escape or cell collapse will occur due to a lack of material strength (cell wall rupture). As a consequence, a curing process taking too long before the gasses are released may result in a hard and dense foam containing very few small cells [19].

The three main steps of a foaming process are nucleation, growth and stabilization of the cellular structure by physical or chemical means [20]. After CFA decomposition or a PBA has been injected, bubbles are produced, which is known as the nucleation step. The bubble nuclei develop into the final bubbles or cells during the growth step. The structure changes due to the development of numerous bubbles, while the cell wall becomes thinner. In order to stabilize the bubbles at a specific size, curing and/or cooling is performed to increase the matrix viscosity. Several physical characteristics, including solubility, viscosity, diffusivity, surface tension and glass transition temperature, have an effect on both processes. Controlling the nucleation and growth steps is very challenging because the foaming and curing processes are carried out simultaneously. To achieve a particular morphology and structure in rubber foams, it is essential to optimize the formulation (foaming agent, matrix type, nanofiller) and processing conditions (pressure, temperature, etc.). The effect of these processing variables is discussed in more detail in the following section.

### 3.1. Foaming Agents

For rubber foam manufacturing, two important families of FA (chemical and physical) are available, with each one having its advantages and disadvantages. Physical foaming agents (PFA) are generally low cost, do not react (inert) and leave no by-products that would influence the general foams’ properties. However, most PFA (besides inert gases) are associated with safety hazards (explosion and flammability), environmental concerns (ozone depletion and greenhouse gases), require specific equipment (high pressure pumps and flow meters) and more complex processes due to their use. This is why, in some cases, it is more appealing to use chemical foaming agents (CFA), which are mostly powders. However, several CFA have health risks despite their widespread use and simple production. Additionally, their application has been constrained by unfavorable by-products and high cost. Nevertheless, CFA is the best, most economical and common choice in the foam industries.

PFA are inert and compressed gas or volatile liquids which are injected into the matrix at high pressure. After heating and depressurization, foaming occurs as a result of gas release or physical state change (liquid to gas). An excellent PFA needs to be thermally stable, nontoxic, odorless and nonflammable, having high solubility, diffusivity and vapor pressure. PFA molecules are divided into two types: inorganic and organic. Nitrogen (N_2_), carbon dioxide (CO_2_), water (H_2_O) and air are the most common inorganic PFA. On the other hand, organic PFA are based on low molecular weight alkane (propane, butane, pentane, hexane), dichloromethane, dichloroethane and some hydrogenated chlorofluorocarbons (HCFC). Moreover, microspheres or micropearls were highly used more recently to prepare foams based on low molecular weight alkanes [21,22], and these materials can be included in the PFA category. Vlentini et al. prepared EPDM foams loaded with various foaming agents, including Expancel (E) and Hostatron (H) [23]. The foaming agent H (mixture of sodium, calcium and potassium bicarbonates, with a decomposition temperature of approximately 150 °C) was used at 1.3 phr, while the foaming agent E (isopentane microspheres, with an expanding temperature above 130 °C) was used at 14 phr to produce EPDM foams. The foaming agent E led to a homogeneous closed cell foam structure with cell sizes of approximately 20 µm, while the foaming agent H produced a mixture of closed/open cells with larger cell sizes (approximately 100 µm) with lower cell size uniformity.

Considering the challenges of using PFA and CFA, another method to produce rubber foams is particle leaching. In this case, water-soluble particles (e.g., potassium or sodium chloride) are added/dispersed in the rubber and then dissolved in a solvent (e.g., water), resulting in a porous cellular structure [24,25]. Although this method has a higher ability to control the cell size and cell density, it still faces several problems such as using a solvent (in compounding and also during leaching), always leads to an open-cell structure, needs high pressure and/or high temperature during compounding, and residual particles can still be present inside the matrix. Peng et al. used sodium chloride (NaCl) as a pore-forming agent inside a SR matrix to produce open cell foams for sound absorption applications [26].

In general, the foaming procedure consists of 4 steps: dissolution, bubble nucleation, bubble growth and curing. The scCO_2_ has a number of advantages over CO_2_ and N_2_ due to its low critical temperature (*T_c_* = 304.15 K) and pressure (*P_c_* = 7.38 MPa), higher solubility and diffusion coefficient [27,28]. One example is ethylene vinyl acetate (EVA) foams produced by Jacobs’s group using scCO_2_ [29]. They reported that increasing the sorption pressure resulted in high density foams with small cell sizes. Similarly, Tessanan and his team showed that increasing the scCO_2_ saturation time and pressure during NR foaming produced foams with smaller cell sizes and narrower cell size distributions [30].

**Figure 3:**
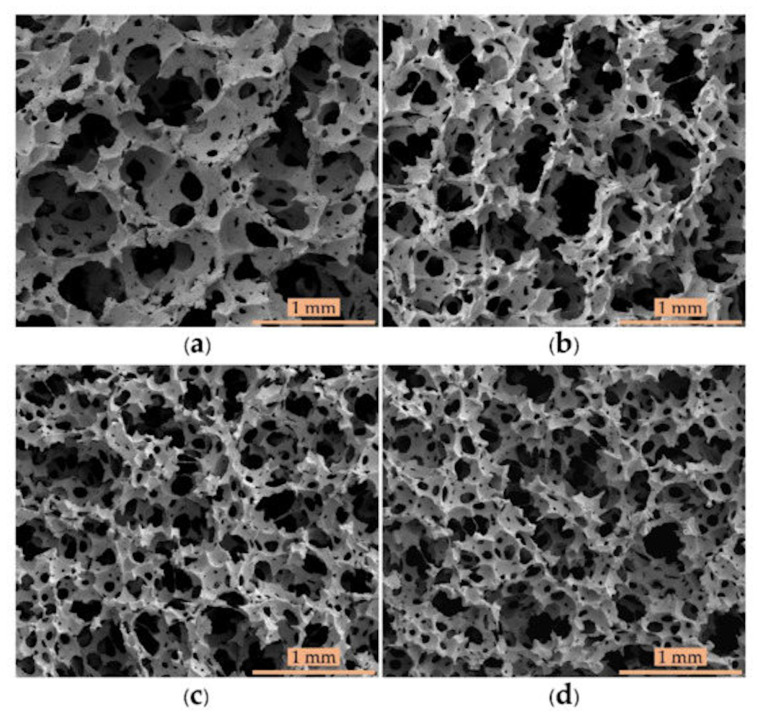
Micrographs of cellular NR based on different CFA contents: (**a**) original formulation, as well as different PO reduction: (**b**) 15%, (**c**) −30% and (**d**) 45% [32].

There are two types of CFA: inorganic and organic. Ammonium, sodium and potassium carbonates, bicarbonates, nitrates and oleate are inorganic CFA. They mostly form open cell morphology in the presence of acids (accelerators) by releasing CO_2_ and water above their thermal decomposition temperature (*T_d_*). The majority of inorganic CFA have a slow endothermic decomposition. A typical dosing for CFA is in the range of 5–10 phr. Most organic CFA reactions are exothermic, which means that they produce energy (heat) above their decomposition temperature. CFA are derived from organic materials such as azodicarbonamide (ADC), 5-phenyl tetrazole (5-PT), *p*-toluenesulfonyl semicarbazide (TSSC), 4,4′-oxybis benzene sulfonyl hydrazide (OBSH) and *N,N′*-dinitroso pentamethylene tetramine (DPT), which produce a gas mixture of ammonia, carbon dioxide, nitrogen and water [31]. Suethao and coworkers produced open cell NR foams with various concentrations of potassium oleate (PO) as a foaming agent [32]. They claimed that decreasing the CFA content by 45% decreased the cell size by 50%, while the cell density was highly improved (800%), as reported in Figure 3.

When a CFA reaches its decomposing temperature, the molecules break down through a sequence of reactions, and the heat generated can activate nearby molecules. This autocatalytic reaction (cascade reactions) leads to a rapid expansion producing closed cells. The ability to control the decomposition temperature (approximately 210 °C for pure ADC) makes ADC an interesting and practical organic CFA. However, several options are available to decrease T*_d_*. For example, decreasing the CFA particle size leads to more surface area for the thermal reaction to occur. Another possibility is to add some activators, such as zinc oxide (ZnO), zinc stearate (ZnSt_2_), stearic acid, etc. However, the addition of ZnO to ADC not only decreases *T_d_*, but also accelerates its decomposition rate [33]. ADC is known to be very efficient (231 cm^3^ of gas/g) [34]. The mechanism of thermal decomposition is directly influenced by temperature and time. However, other factors, including the type, amount and particle size of the activators and CFA, as well as their level of dispersion, affect the thermal decomposition. CFA generate decomposition residues acting as nucleation agents for subsequent foaming, leading to a finer porous structure. Nevertheless, CFA can also have a detrimental impact on the material’s properties or affect the color of the final product [35]. For example, the density of POE foams was reported to be mainly controlling the physical and mechanical properties [36]. As a result, increasing the ADC content from 1 to 13 phr decreased the density (820 to 57 kg/m^3^), being less resilient (84–23%) with lower tensile strength (5.25 to 0.82 MPa) and elongation at break (602–127%). This trend was related to a higher gas volume resulting in lower mechanical properties.

### 3.2. Matrix

**Figure 4:**
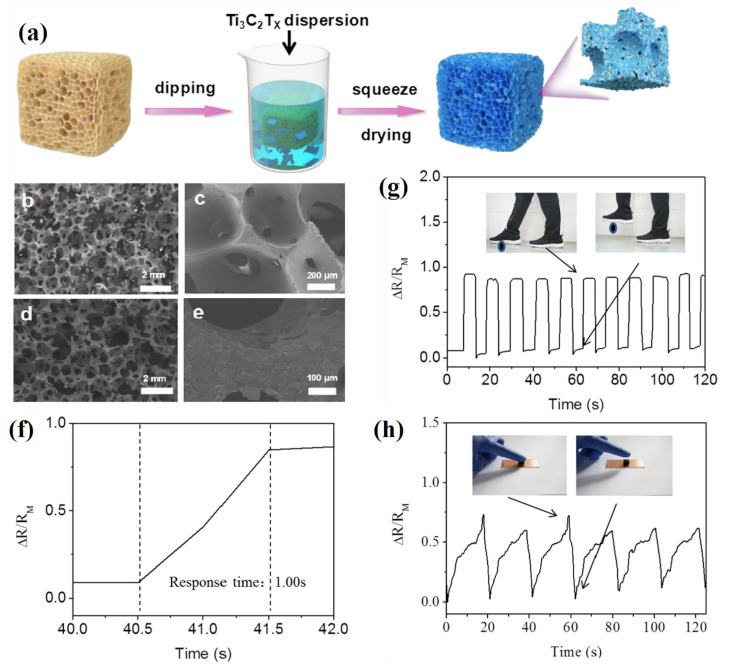
(**a**) The preparation steps for NR/MXene foams. (**b**,**c**) Micrographs of NR foams, (**d**,**e**) SEM images of NR/MXene foams, (**f**,**g**) the partial amplified signal (response time) and step monitoring from an adult and (**h**) a finger pressing application of the MXene/NR foam [38].

Rubbers such as NR, EPDM, SBR, SR, etc., are the main matrices for rubber foams. Salmazo and coworkers investigated the effect of modified and unmodified NR matrix [37]. The modified compound was produced by epoxidized natural rubber (ENR). Both foams were prepared with ADC (10 phr) and cured under a range of electron beam irradiation doses, from 50 to 150 kGy. ENR foams were found to have higher nucleation rates and minimal cell degradation compared with foams made from NR. This was explained by the fact that the cured ENR foam included epoxide groups, which increased the curing level. By using a dip-coating technique, a low-cost and highly sensitive strain sensor of titanium carbide (MXene)/NR foam was prepared by assembling the Ti_3_C_2_T_x_ nanosheets on the side and surface of NR foams (Figure 4a–e) [38]. According to the findings, the prepared smart materials showed extremely high sensitivity (GF = 14) to very low mechanical solicitation (low detection limit of 435 Pa), showing excellent adhesion between MXene and NR foam. In addition, the MXene/NR strain sensors exhibited a wide strain range from 0 to 80% and a low response time of 1 s (Figure 4f), which is of high importance to design wearable electronics, as the materials can detect a wide range of actions such as step monitoring and finger pressing (Figure 4g,h).

Lee and colleagues produced nanocomposite foams based on EPDM filled with halloysite nanotube (HNT) [39]. A batch reactor was used and the FA was scCO_2_. They generated foams with high cell density (1.5 × 10^10^ cell/cm^3^) and low cell size (7.8 µm). In this case, HNT were shown to be effective nucleating agents. Shao et al. reported a double crosslinking system by adding sulfur and dicumyl peroxide (DCP) at the same time for SBR (styrene–butadiene rubber) foaming [40]. The synergetic effect between both curing agents led to lower shrinkage (2.25%). The effect of single wall carbon nanotube (SWCNT) in EPDM/SBR foams was studied by Bahadar’s group [41]. The incorporation of SWCNT (0.6% wt.) inside the EPDM/SBR blend increased the storage modulus (80%) while decreasing the loss modulus (27%).

In order to improve the characteristics (compression set, rebound resilience and tensile strength) of ethylene vinyl acetate (EVA) foams, ethylene-*1*-butene (EtBC) was added [42]. By combining NR with EVA, Kim et al. were able to produce foams having good tear strength and high rebound resilience at low density using the optimum temperature for curing [43]. The effect of the EVA:CPE ratio on the foaming and curing steps leading to improved mechanical characteristics was also investigated [44]. It was found that adding more EVA had no effect on the kinetics (curing time and scorch time). On the other hand, the ratio had a direct effect on hardness, rebound resilience and shrinkage. It was also observed that the cell density decreased for an EVA:CPE ratio from 0:100 to 50:50, but significantly increased from 30:70 to 10:00. Overall, the ER (void content) increased with increasing EVA content.

**Figure 5:**
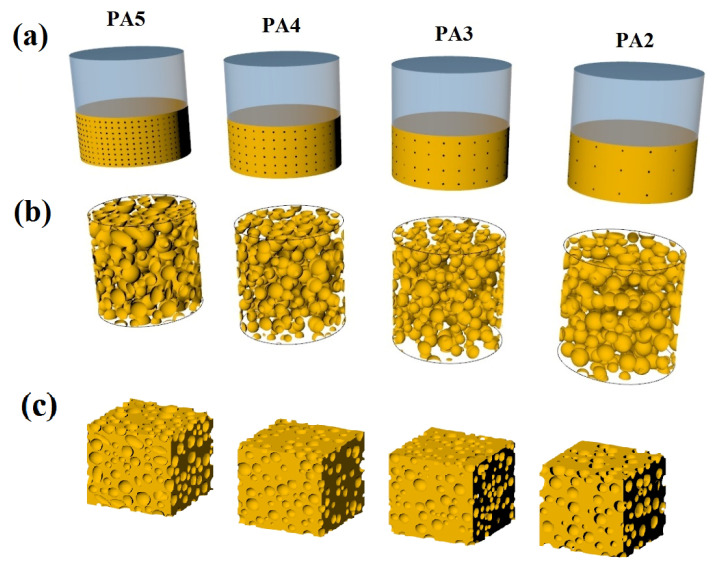
Schematic representation of the foaming process of POE with various ADC concentrations (2–5 phr, from right to left): (**a**) nucleation, (**b**) final structure in the mold and (**c**) final foam structure [47].

The viscoelastic properties of SR foams made with scCO_2_ were examined by Liao et al. with respect to silica content, saturation temperature (*T_s_*) and saturation pressure (*P_s_*) [45]. They observed that because silica acted as a heterogeneous nucleation agent and viscosity enhancer, its concentration had a very important effect on both cell nucleation and cell growth. Moreover, by lowering the *T_s_* (from 80 to 40 °C), cell nucleation was important because the cell density was increased (0.4–9.0 × 10^6^ cells/cm^3^) while the cell diameter was decreased (76 to 21 μm). Conversely, higher *T_s_* (60 and 80 °C) induced cell rupture and coalescence associated with rapid cell growth (lower viscosity and elasticity). Finally, increasing *P_s_* (from 10 to 14 MPa) increased the plasticization effect of scCO_2_ and reduced the viscosity of the SR matrix, which again accelerated the cell growth rates. To investigate the relationship between cell sizes and mechanical properties, Luo et al. produced SR foams using spherical urea with various sizes (200–800 μm) as cell-forming agents [46]. They showed that increasing the cell size from 200–300 μm to 600–800 μm resulted in higher compressive strength from 0.257 MPa to 0.382 MPa. Because of the larger cell size (higher amount of gas volume), this improved the resistance of SR foams against compressive forces. On the contrary, the tensile results showed that increasing the cell size from 200–300 μm to 600–800 μm reduced the elongation at break (156–110%) and tensile strength (0.667 MPa to 0.433 MPa). These trends are expected since larger cell sizes generate higher stress concentration points, leading to lower tensile properties. In another work, POE foams were produced using different CFA contents (ADC: 2–5 phr) without any curing agent [47]. The cell size decreased by 29% and the cell density increased by 470% as the ADC content increased (2 to 4 phr), as reported in Figure 5. However, due to cell coalescence, 5 phr ADC produced bigger cells (148 μm) combined with less cell density (483 cells/mm^3^). However, increasing the ADC content (2 to 5 phr) was found to decrease all the tensile properties: elongation at break (16%), strength at break (21%) and modulus (28%), as well as hardness (14%). In addition, a finite element method (FEM) was used to simulate the tensile behavior with good precision.

### 3.3. Nanofillers

Filler addition is always a starting point to improve the mechanical properties of any material. This is especially important for elastomers since the neat matrix usually has very low modulus and strength [48]. However, the addition of fillers modifies crosslinking parameters such as curing and scorch times, with both having a direct effect on the aging (stability) and mechanical properties. Rubber foams have been reinforced using a variety of fillers, but the most common ones are carbon black (CB), NG, CNT, nanofibers, NC and nanosilicates [3,49,50,51]. Several parameters must be determined to quantify the improvements, including the particle size and geometry, the dispersion level, aspect ratio and orientation. Other parameters are the chemical or physical bonding (type and number) with matrix molecules [52].

**Figure 6:**
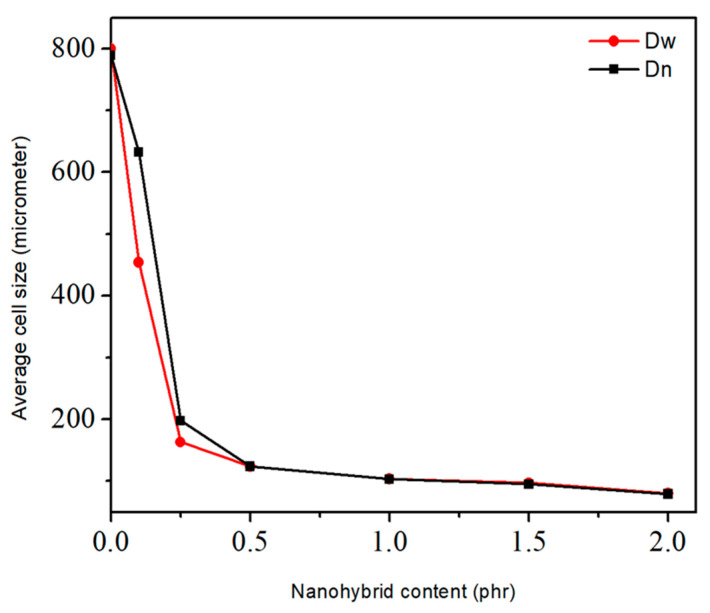
Average cell size of hybrid nanocomposite foams with respect to different CNT/GNS concentration [48].

When CNT was replaced by NG (50:50 hybrid nanofiller), it was shown that the CNT/NR morphology changed from a discontinuous set of agglomerates into a continuous 3D network [53]. The thermal stability of EPDM foams was found to be enhanced with increasing CB concentration [54]. The viscoelasticity and foaming behavior of EPDM foams were reduced, while the curing and mechanical strength were enhanced. Vahidifar et al. developed a hybrid reinforcing system including NC and CB to study the effect of NC concentration (0–10 phr) on the crosslinking, morphological and mechanical performances of NR foams containing CB (10 phr) [10]. A rheological analysis confirmed that higher NC concentration reduced the scorch time and curing time (up to 50%). The tensile modulus and hardness of the compounds increased as the NC concentration increased, but the foam’s resilience and elongation at break decreased. The production of foams with more uniform and smaller cells was easier by increasing the NC concentration from 0 to 5 phr. On the other hand, adding 7 phr NC resulted in a foam morphology made of two sections having various cell diameters and even producing a bimodal distribution. scCO_2_ was also used to prepared SR/silica foams (saturated at 22 MPa and 50 °C for 1 h) to study the effect of silica concentration (40–70 phr) on the cellular morphology of microcellular closed cell SR foams [55]. According to the morphological results, the SR foam with 50 phr silica exhibited a maximum value of cell size (1.82 µm) and a minimum value of cell density (2.41 × 10^10^ cells/cm^3^). However, higher silica content (70 phr) generated higher cell density (1.02 × 10^11^ cells/cm^3^). These results indicate that the presence of silica is highly important to improve the heterogeneous nucleation of the cells, as well as acting as a reinforcing agent. To improve the mechanical and morphological characteristics of NR foams, Shojaei and colleagues used a physical hybrid system of NG and carbon nanotubes (CNT) [48]. As a result, increasing both CNT and GNS concentration from 0 to 2 phr decreased the cell diameter (800–80 μm in Figure 6) and improved the stress (50% deformation) from 150 to 440 kPa.

**Figure 7:**
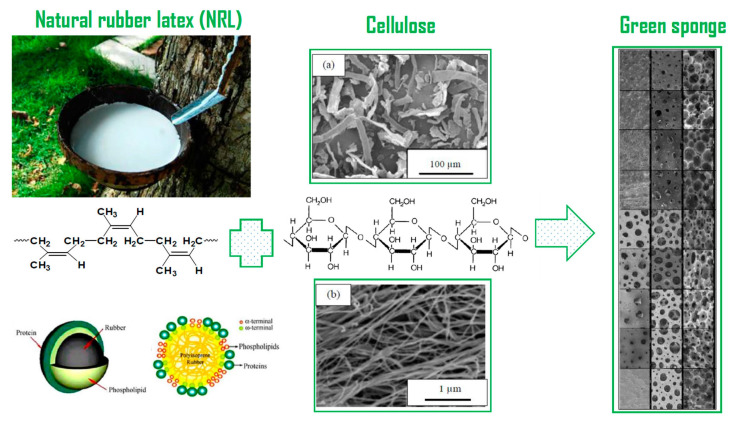
The structure and SEM images of (**a**) MC fibers and (**b**) NC fibers, as well as their green NRLF foams [57].

Pongmuksuwan and coworkers produced flexible electromagnetic absorber foams based on NR and CB/Fe_3_O_4_ as fillers [56]. Higher CB content (4–10 phr) led to lower average cell size (780–670 µm). A similar trend was observed for Fe_3_O_4_ addition. Compared to a foam containing Fe_3_O_4_, the foams produced with CB had higher tensile strength and modulus. This was because CB has some functional groups (hydroxyl or carboxyl groups) which improved its compatibility and interactions with NR chains. For example, the modulus of NR/CB and NR/Fe_3_O_4_ foams (10 phr of filler) gave 0.0015 MPa and 0.0007 MPa, respectively. Furthermore, the addition of CB (4–10 phr) increased the compression set by 25%. This showed that higher CB concentration decreases the recoverability of these foams. On the other hand, the incorporation of Fe_3_O_4_ did not have a significant effect on the compression set because of its limited interaction with NR chains. Hence, the compression set of NR/Fe_3_O_4_ increased from 18% to 26% by increasing Fe_3_O_4_ from 4 to 10 phr due to its rigidity which decreased the NR foams’ elasticity. Phomark and coworkers used microcellulose (MC, 10–100 μm) and nanocellulose (NC, 10–3000 nm) fibers (5–20 phr) to prepare green and open cell NRLF (cell size: 10–500 μm) in the presence of PO as CFA (Figure 7) [57]. NC was found to have a better dispersion inside the NRLF matrix leading to better reinforcement compared to MC. By adding 5 phr MC or 15 phr NC, the highest tensile strengths were 0.43 MPa and 0.73 MPa, respectively. Compression tests also revealed that 15 phr NC increased the recovery to 34.9% (1.3-fold improvement), while no significant improvement was observed for MC-NRLF.

**Figure 8:**
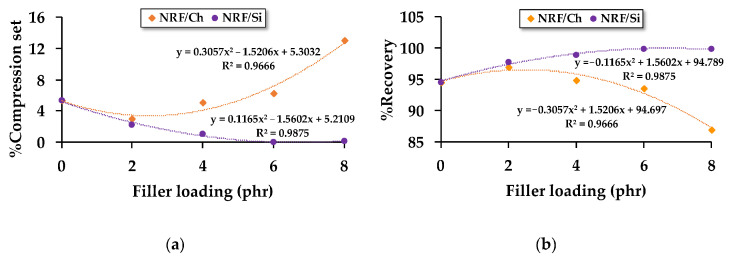
Compression properties of NR foams based on different types and concentrations of fillers (charcoal and silica): (**a**) compression set and (**b**) recovery [58].

Different fillers, such as charcoal and silica (2–8 phr), were incorporated into NR to investigate the recovery properties of their foams [58]. Increasing the charcoal content led to larger cell size and lower cell density (Table 1), while the cell density increased and the cell size decreased with higher silica concentration. In contrast to charcoal, the recoverability of NR foam was improved (low compression set) as the silica content was increased (Figure 8). Hence, the ability for NR foams having charcoal concentrations above 2 phr to return to their original shape was reduced when compressed at 75% of their original thickness for 72 h. As the compression set decreased, elasticity increased. Thus, NR foams filled with silica showed higher elasticity than NR foams containing charcoal. This is as a result of silica’s higher specific surface area having better filler–rubber interaction than charcoal.

In another work, Prasopdee and colleagues used cassava starch (Cs) as a low density filler (lower than charcoal and silica) to produce NR foams [59]. The foam density of NR/Cs was decreased compared to NRF filled with charcoal (14–16%) and silica (18–28%). The addition of Cs (4–12 phr) slightly decreased the cell size (0.54 to 0.52 mm) while improving the cell density (11,540 to 12,319 cells/cm^3^). Although the cellular properties of the foams did not substantially change with increasing Cs content, the compression strength did increase (6.1 to 10.5 kPa). This behavior was related to higher crystallinity of Cs and chemical reactions between starch and vulcanization agents leading to higher stiffness.

### 3.4. Foaming and/or Saturation Temperature

Cell size and cell density (foam density) are directly impacted by the viscosity, which is mostly controlled by temperature [60]. While cell expansion requires high viscosity (low temperature) for easier nucleation, efficient foaming requires low viscosity (high temperature) for easier expansion. The formation of a cellular structure represents an equilibrium between two processes. First, the amount and kinetics of the gas generated from the FA decomposition acting as the blowing force leading to high bubble nucleation and growth. The second effect is an increase in the matrix viscosity related to the crosslinking reaction restricting further expansion and leading to stabilization. As a result, the interaction between the curing and foaming processes can help to identify the ideal foaming temperature. The relationship between NR foams’ physical and mechanical characteristics and the foaming temperature (145–155 °C) was studied by Kim and coworkers [61]. As the foaming temperature was increased, the NR density decreased. Because the cell density within the rubber matrix increased with temperature, using lower temperatures (145 and 150 °C) decreased the densities compared to a higher temperature (155 °C). They also observed that for these NR foams, 155 °C was the optimum temperature for vulcanization and foaming. Because of the lower density, it was observed that the tensile strength decreased with increasing temperature, indicating higher foaming effectiveness. Additionally, the tensile modulus and tear strength both continuously decreased as the temperature increased.

**Figure 9:**
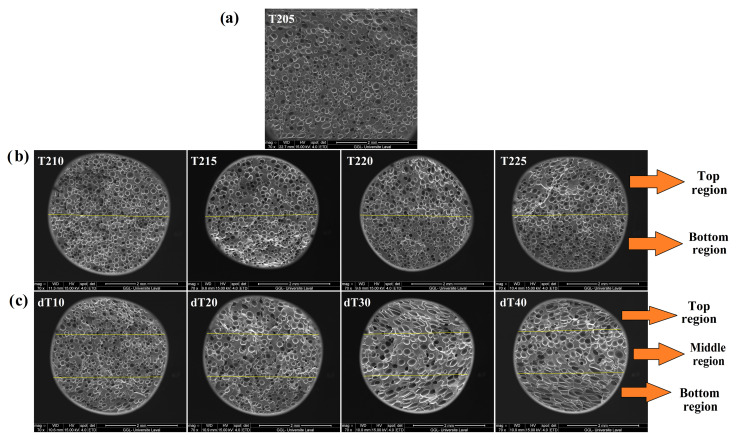
SEM images of: (**a**) uniform and graded POE foam at: (**b**) different T_avg_ of 207.5, 210, 212.5 and 215 °C (from left to right) and (**c**) different ΔT of 10, 20, 30 and 40 °C (from left to right) [15].

Liu’s group produced SR/silica (70 phr) foams at different *T_s_* (50–80 °C) using scCO_2_ (*P_s_* of 22 MPa for 1 h) [55]. They observed that the cell size of foams saturated at 80 °C was 6.5 times compared to 50 °C. This was due to the lower sorption of CO_2_ molecules caused by increasing *T_s_* leading to lower nucleation spots and higher cell growth inside the matrix. Furthermore, a higher *T_s_* decreased the elasticity/viscosity of the SR matrix leading to higher cell coalescence. According to Najib and colleagues, increasing the temperature generates higher gas pressure, stretching the cell walls even more [62]. This high cell wall expansion resulted in a substantially larger cell structure and a reduced foam density. Thus, there was less crosslinking and less solid phase (per unit volume). The cell size distribution (CSD) was improved, while the range of cell sizes was higher by increasing the foaming temperature. Compared to foams made at 150 °C and 160 °C, the foams made at 140 °C showed a more homogeneous CSD. Rostami et al. achieved functionally graded POE foams by changing the processing temperature (molding temperature), the average temperature (*T_avg_*) and temperature difference (Δ*T*), as shown in Figure 9 [15]. By tailoring the cell size and cell density (across thickness), different thermal conductivities (0.125–0.180 W/m.K) were obtained. Increasing *T_avg_* (from 207.5 to 215 °C) decreased the tensile properties, including the modulus, strength and elongation at break by 33%, 13% and 15%, respectively. On the contrary, increasing Δ*T* (from 10 to 40 °C) improved these properties by 14%, 26% and 10%, respectively. According to Pechurai and colleagues, raising the processing temperature reduced the scorch and cure times since the curing processes were faster [63]. Due to the direct effect of thermal breakdown on the heat produced (exotherm) or consumed (endotherm), which affects the crosslinking properties, the optimum curing temperature for a specific rubber matrix is not always the same, with or without CFA.

### 3.5. Foaming and Saturation Pressure

**Figure 10:**
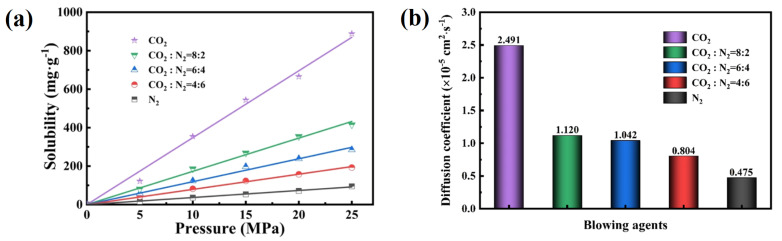
(**a**) Solubility as a function of gas pressure (50 °C) and (**b**) diffusion coefficient of the different PFA in SR foam at 50 °C and 10 MPa [66].

The amount of pressure applied when forming or molding a sample has a significant effect on how the rubber foam’s morphology will develop. Kim and coworkers investigated the effect of foaming pressure on the structure and properties of NR foams [64]. Higher density (lower ER) and decreased foaming efficiency were produced when the foaming pressure was increased. With increasing foaming pressure, the hardness and tear strength steadily increased, but lower elongation at break was observed. scCO_2_ is considered as the best PFA because it is an eco-friendly alternative and its *P_s_* was shown to have a significant effect on the cellular microstructure. Moreover, using scCO_2_, Tessanan and associates investigated different pressures (0–12.5 MPa) to determine the effect on NR foams’ morphology [30]. According to their findings, increasing *P_s_* decreased the cell diameter (below 10 µm) and narrowed the CSD. The main reason is that higher pressure decreases the activation energy for nucleation and more gas molecules (higher saturation/solubility) are available to produce the nuclei. A number of high temperature vulcanizates (HTV) SR microcellular foams were generated based on CO_2_ [65]. Increasing *P_s_* improved the CO_2_ diffusivity in the HTV silicone rubber as well. Additionally, increasing the *P_s_* made the matrix absorb more CO_2_, leading to smaller cell diameter and higher cell density (higher gas concentration). Hence, Xiang and coworkers studied the effect of various *P_s_* (16–22 MPa) of scCO_2_ on the morphological properties of SR/silica (70 phr) foams prepared at 50 °C for 1 h [55]. Cell size decreased (2.11–0.708 µm), while the cell density increased (0.296–1.02 × 10^11^ cells/cm^3^) if *P_s_* increased (16–22 MPa). According to Xiang’s group, it can be concluded that both *T_s_* and *P_s_* have a different effect on the morphological properties of rubber foams. Increasing *P_s_* leads to higher cell density and lower cell size, while increasing *T_s_* results in lower cell density and higher cell size in SR foams produced by scCO_2_. Wang et al. investigated the solubility of different PFA (CO_2_, N_2_ and their mixture) and the morphology of SR foams [66]. Their results showed that for a single PFA, increasing the pressure increased their solubility in SR (Figure 10a). On the other hand, the CO_2_ diffusivity and solubility were higher compared to N_2_ under similar conditions (Figure 10b). They also observed that changing the CO_2_:N_2_ ratio can be used to modify the solubility and diffusion coefficient of the blowing agent system. In particular, a CO_2_:N_2_ ratio of 4:6 produced a highly uniform cellular structure with a foam density as low as 0.086 g/cm^3^.

Zhu et al. proposed a novel scCO_2_ aided post-vulcanization method to develop microcellular epoxidized natural rubber foam (f-ENR) with enhanced ductility, durability and cellular structure stability [67]. They observed that when *P_s_* increased from 15 MPa to 25 MPa, the cell diameter decreased (1.70–1.30 µm) while the cell density increased (1.10 × 10^11^–1.40 × 10^11^ cells/cm^3^). However, their results showed that when the *T_s_* was raised from 35 °C to 75 °C at 15 MPa, the cell density decreased (1.10 × 10^11^–8.40 × 10^9^ cells/cm^3^) while the cell diameter increased (1.70–5.50 µm). These trends were attributed to the decreasing solubility of CO_2_ in the ENR matrix when the *T_s_* increases, thus limiting cell nucleation.

## 4. Conclusions

### 4.1. General Conclusions

Rubber foams are lightweight materials having a number of favorable characteristics, including low density, high elasticity and flexibility, high tear strength and great resistance to abrasion, as well as good thermal and acoustic insulation. These properties make rubber foams ideal for civil and industrial applications, with examples including automotive, aerospace, insulation, medical, construction, etc. Depending on the formulation, processing technique and foaming conditions used during their production, rubber foams can have different morphology (open or closed cell) with cell size ranging from millimeter to nanoscale. Table 2 presents a summary of the materials, foaming process parameters and physical and morphological properties of the studies included in this review paper.

Three steps are important in the production of rubber foams: nucleation, growth and stabilization. Initially, gas nuclei are formed by a foaming agent (chemical or physical) inside the rubber matrix. These cells then grow in size as the curing proceeds to stabilize the morphology. Two important factors have an effect on the morphological, mechanical, physical and thermal properties. The first parameter is associated with formulation, while the second is the conditions used for processing. Type of rubbers, accelerators, foaming agents and fillers, along with their quantities, are associated with composition, while the steps of foaming (pressure, temperature, time, etc.) and crosslinking (pre-curing process, curing temperature and time) are associated with the production conditions. Most of the studies investigated how these factors affect the characteristics of rubber foams and they have been reviewed in this work. To fully understand all the relations between formulation, processing, structures and characteristics, further research is still required, as described next.

### 4.2. Opening for Future Work

The majority of the work performed in the early years of foam manufacture was devoted to developing different porous morphologies. Based on the development in nanotechnology, the focus switched to various nanoparticles and how they affect the characteristics of cellular polymeric materials. The study on nanocomposite rubber foams is still in its early stages and more work is needed to determine how the rubber foam’s structure and properties are affected by the size, shape and surface chemistry of these nanoparticles. The theory of rubber foaming is still not completely understood, as the processes of foaming and curing are interrelated and very complex since they depend on several factors, such as pressure, temperature, concentration, time and pre/post-curing conditions, as well as the rubber type (single resin or blends). Finally, more work should be devoted to the modeling and simulation of all the steps involved in rubber foaming, including cell nucleation and growth in a rheologically complex multiphase material under unsteady and non-isothermal conditions.

## Figures and Tables

**Table 1:** Morphological properties of NR foams with different types and concentrations of fillers (charcoal and silica) [58].

Sample Name	Average Cell Size (μm)	Cell Density (Cells/cm^3^)
NRF (unfilled)	836	3041
NRF/2 Ch	860	2778
NRF/4 Ch	988	1807
NRF/6 Ch	1081	1379
NRF/8 Ch	1092	1333
NRF/2 Si	1079	1412
NRF/2 Si	909	2316
NRF/2 Si	876	2546
NRF/2 Si	673	5606

**Table 2:** Summary of the materials, foaming process parameters and physical and morphological properties of rubber foams.

Matrix	Foaming Agent	Filler	Foaming Process	Density (g/cm^3^)	Morphology	Reference
NR	ADC (4 phr)	NC (0–10 phr)	T (155 °C) P (50 bar) t (30 min) *	0.40	Closed cell CS (340–143 μm) CD (35–225 cells/mm^3^) **	[10]
EPDM	OBSH (3 phr)	CNT (1–3 phr) NC (2–6 phr)	T (160 °C) P (50 bar) t (20 min)		Closed/open cell CD (5.21 × 10^10^–6.28×10^10^ cells/cm^3^)	[11]
EPDM	Micropearl (14 phr) Expancel (14 phr) Sodium chloride (305 phr)	CB (20 phr)	T (170 °C) P (2 bar) t (20 min)	0.23–0.42	Closed/open cell CS (60–80 μm)	[12]
POE	ADC (4 phr)		T_avg_ (205–215 °C) ΔT (0–40 °C) P (8.5 bar) T (12 min)	0.55–0.72	Closed cell CS (98–337 μm) CD (204–820 cells/mm^3^)	[15]
SR	scCO_2_	Silica (26 wt.%)	T (40–70 °C) P (10–15 MPa) t (1 h)	0.25–0.70	Closed cell CS (5–266 μm) CD (2.60 × 10^5^–5.60 × 10^9^ cells/cm^3^)	[16]
NR	NaHCO_3_ (3 to 12 phr)		T (150 °C) t (45 min)		Closed cell CS (420–620 μm)	[17]
SR		NG (1–4 wt.%)	T (60 °C) t (1 h)		Closed/open cell CS (191.00–79.40 μm) CD (2.13 × 10^5^–7.15 × 10^5^ cells/cm^3^)	[18]
EPDM	Expancel (14 phr)		T (170 °C) P (2 bar) t (10 min)	0.48–0.59	Closed cell CS (20 μm)	[23]
EPDM	Hostatron (1.3 phr)		T (170 °C) P (2 bar) t (10 min)	0.57–0.73	Closed cell CS (100 μm)	[23]
SR	NaCl (0–100 phr)		T (150 °C) t (20 min)	0.44–0.63	Close/open cell CS (100–300 μm)	[26]
NR	PO (0–45%)		T (90 °C) t (2 h)	0.11–0.12	Open cell CS (273–548 μm) CD (10,241–82,450 cells/cm^3^)	[32]
POE	ADC (1–13 phr)		T (170 °C) P (10 MPa) t (10 min)	0.06–0.82	Closed cell CD (3.79 × 10^9^–6.29 × 10^10^ cells/cm^3^)	[36]
NR	ADC (10 phr)		Electron beam irradiation (50–150 kGy) t (30–90 s)		Closed cell CS (22.60–74.72 μm) CD (4.92 × 10^5^–249.60 × 10^5^ cells/cm^3^)	[37]
ENR	ADC (10 phr)		Electron beam irradiation (50–150 kGy) t (30–90 s)		Closed cell CS (6.83–74.57 μm) CD (4.19 × 10^5^–1975.20 × 10^5^ cells/cm^3^)	[37]
EPDM	scCO_2_	HNT (0–10 phr)	T (130 °C) P (30 MPa) t (24 h)	0.23–0.35	Closed cell CS (7.80–12.00 µm) CD (4.21 × 10^9^–1.54 × 10^10^ cells/cm^3^)	[39]
SBR	ADC (3 phr)		T (200 °C)	0.22–0.83	Closed/open cell CS (7.68–22.94 μm)	[40]
SR	scCO_2_	Silica (30–50 phr)	T (40−80 °C) P (10−14 MPa) t (1 h)		Closed/open cell CS (4.24–77.10 µm) CD (0.40 × 10^6^–1.40 × 10^9^ cells/cm^3^)	[45]
POE	ADC (2–5 phr)		T (205 °C) P (8.5 MPa) t (12 min)	0.61–0.75	Closed cell CS (109.40–153.10 µm) CD (103–591 cells/mm^3^)	[47]
NR	ADC (6 phr)	CNT/NG (0–2 phr)	T (160 °C)		Closed cell CS (80–800 μm)	[48]
SR	scCO_2_	Silica (40–70 phr)	T (50–80 °C) P (16–22 MPa) t (1 h)		Closed cell CS (0.71–4.60 µm) CD (0.40 × 10^10^–1.02 × 10^11^ cells/cm^3^)	[55]
NR	PO (1.5 phr)	Microcellulose/nanocellulose (5–20 phr)	T (100 °C) t (1 h)	0.15–0.31	Open cell (10–500 μm)	[57]
NR	PO (3.63%)	Cs (4–12 phr)	T (90 °C) t (2 h)	0.069–0.071	Open cell CS (0.54–0.52 μm) CD (11,540–12,319 cells/cm^3^)	[59]
ENR	scCO_2_		T (35 °C) P (15–25 MPa) t (1 h)		Closed cell CS(1.30–1.70 µm) CD (1.10 × 10^11^–1.40 × 10^11^ cells/cm^3^)	[67]
ENR	scCO_2_		T (35–75 °C) P (15 MPa) t (1 h)		Closed cell CS (1.70–5.50 µm) CD (8.40 × 10^9^–1.10 × 10^11^ cells/cm^3^)	[67]

* T—temperature, P—pressure, t—time. ** CS—cell size, CD—cell density.

## Data Availability

Not applicable.
